# Impact of dual residual risk of cholesterol and inflammation on adult male sex hormones: a cross-sectional study from NHANES

**DOI:** 10.3389/fendo.2025.1526056

**Published:** 2025-03-10

**Authors:** Yang Zhou, Guofeng Wang, Li Liu, Jie Yu, Shiying Ju

**Affiliations:** ^1^ Department of Urology, Sunshine Union Hospital, Kuiwen, Weifang, China; ^2^ Department of Anesthesiology, Weifang People’s Hospital, Kuiwen, Weifang, China; ^3^ Department of Rheumatology Immunology, Sunshine Union Hospital, Kuiwen, Weifang, China; ^4^ Department of radiology, Weifang People’s Hospital, Kuiwen, Weifang, China

**Keywords:** testosterone, inflammation, residual cholesterol, residual risk, testosterone deficiency

## Abstract

**Purpose:**

Sex hormones are closely linked to inflammation and lipid metabolism. This study explores the correlation of residual cholesterol risk and residual inflammation risk with sex hormones.

**Materials and methods:**

Logistic regression and dose-response curve analyses were conducted to examine the associations of total testosterone (TT), Sex Hormone Binding Protein (SHBG), Estradiol (E2), and Free testosterone (FT) with low density lipoprotein cholesterol (LDL-C) and high sensitive c-reactive protein (hs-CRP). Testosterone deficiency, defined as TT below 300 ng/dL, was analyzed across various subgroups based on LDL-C and hs-CRP levels. Grouped by LDL-C and hs-CRP: normal, LDL-C < 2.6 mmol/L, hs-CRP < 3mg/L, residual cholesterol risk only (RCR): LDL-C ≥ 2.6 mmol/L, hs-CRP < 3mg/L, residual inflammation risk only (RIR): LDL-C < 2.6 mmol/L. hs-CRP ≥ 3mg/L, both risk (BR): LDL-C ≥ 2.6 mmol/L, hs-CRP ≥ 3mg/L.

**Results:**

The results indicated a negative association between hs-CRP and TT (β = -1.98, 95% CI [-3.54, -0.42], p = 0.013), as well as FT (β = -0.04, 95% CI [-0.07, -0.02], p = 0.0002). Similar trends were observed for the relationship between hs-CRP and SHBG (β = -3.61, 95% CI [-5.33, -1.90], p = 0.0003). In the presence of both risk factors (BR), TT decreased most significantly (β = -79.37, 95% CI [-112.74, -46.00], p < 0.0001), as did FT in the same subgroup (β = -1.00, 95% CI [-1.61, -0.40], p = 0.0012). Notably, hs-CRP exhibited a non-linear correlation with TT, SHBG, and FT, with distinct inflection points. Furthermore, in diabetic patients, hs-CRP was positively linked to E2 (β = 0.39, 95% CI [0.03, 0.74], p = 0.0328).

**Conclusions:**

LDL-C was independently correlated with SHBG, hs-CRP with TT and FT, and the BR population had a higher risk of testosterone deficiency. Special populations with diabetes and hypertension need to be concerned about residual cholesterol risk and inflammatory risk.

## 1 Introduction

Testosterone is a hormone that plays a key role in the development and maintenance of male reproductive tissues, such as the testes and prostate, and secondary sexual characteristics, such as muscle mass, bone density, and body hair. Testosterone is synthesized in the Leydig cells of the testes and is transported in the bloodstream in combination with a carrier protein called sex hormone binding globulin (SHBG) (1).

SHBG is produced in the liver and binds highly affinity to testosterone and other sex hormones such as estradiol. By binding to these hormones, SHBG regulates their bioavailability and distribution in the body, which can affect their biological activity and influence a wide range of physiological processes (2). Studies have also shown that abnormal testosterone levels may be associated with a number of non-reproductive disorders, such as cardiovascular disease, metabolic disorders and cognitive impairment (3). For example, low testosterone levels are associated with an increased risk of developing type 2 diabetes, obesity and metabolic syndrome, while high levels of SHBG are associated with a reduced risk of developing cardiovascular disease, and a low-fat diet also increases testosterone levels (4, 5).

Residual cholesterol represented by low density lipoprotein cholesterol (LDL-C) and residual inflammatory markers by high sensitive-c-reactive-protein (hs-CRP) have been shown to be high risk factors for cardiovascular events, and even after receiving aggressive therapeutic medications, there is still an increased risk of cardiovascular disease recurrence with elevated LDL-C. Sex hormones are strongly associated with lipid metabolism and inflammation, with one study showing a significant negative correlation between LDL-C and testosterone and SHBG (6), and that androgen therapy also significantly improves lipid levels and increases LDL-C (7). The effect of hs-CRP and sex hormones was demonstrated in a survey of 12-16 year old adolescents in which hs-CRP was negatively correlated with testosterone (8) and SHBG was independently and negatively correlated with hs-CRP concentrations in males (9).

Both LDL-C and hs-CRP are strongly associated with sex hormones, then more studies are needed to demonstrate the relationship between these two residual risks and sex hormones and the extent to which these two wind residual risks jointly affect sex hormones.

In the current study, we analyzed the relationship between sex hormones and dual residual risk of cholesterol and inflammation in adult men using a population from the NHANES database.

## 2 Materials and methods

### 2.1 Study population

**Figure 1:**
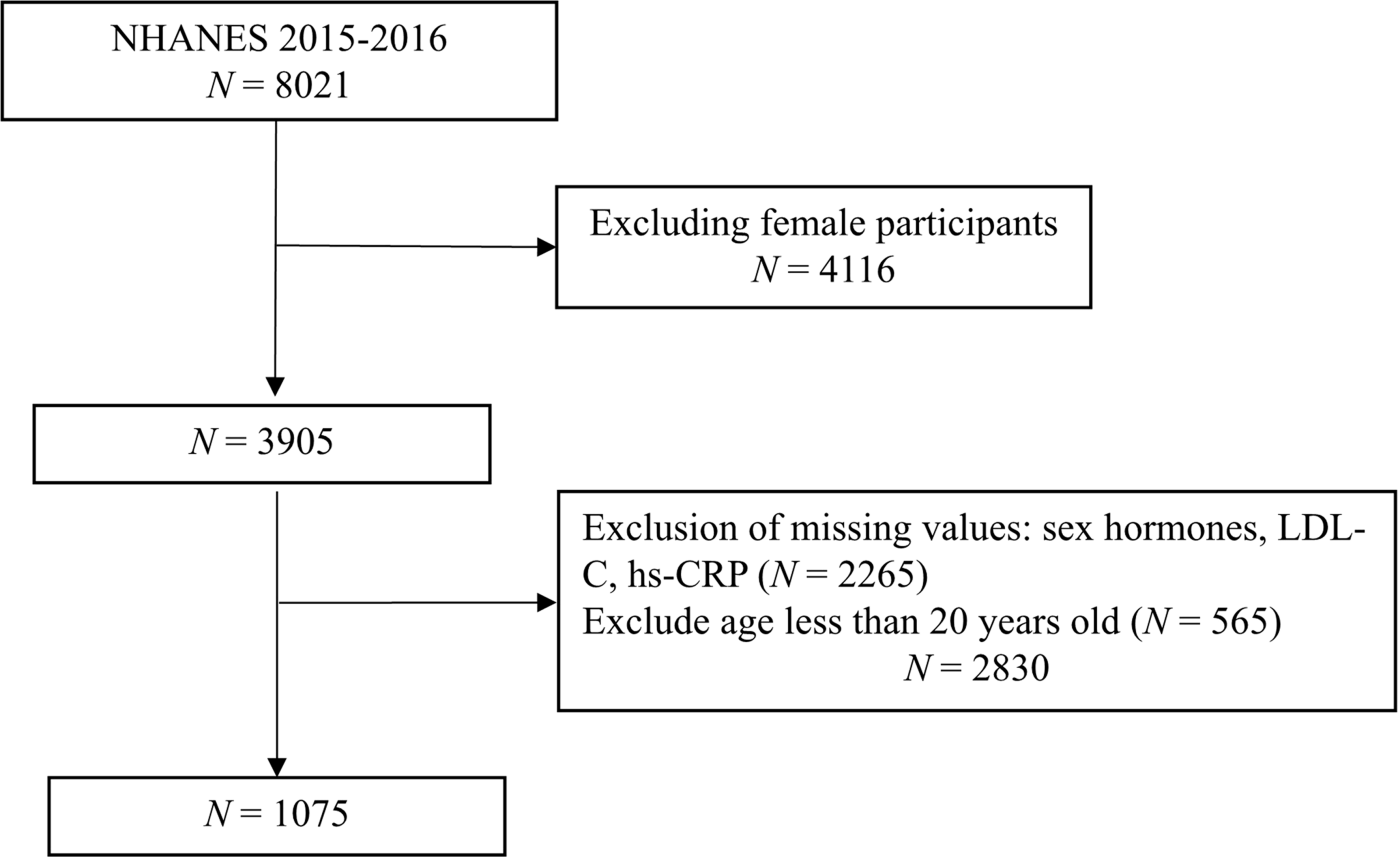
Flow chart.

We conducted an analysis of the physical status of the US population using data from the National Health and Nutrition Examination Survey (NHANES), conducted by the Centers for Disease Control and Prevention (CDC). The survey used a multistage, stratified, whole group survey design to ensure representation of the non-institutionalized population in the US. All NHANES participants gave written informed consent and the study was approved by the NCHS Institutional Review Board. For this cross-sectional study, we included men aged 20 years and older from the 2015-2016 survey cycle and calculated survey weights. We removed extreme values and covariates with more than 10% missing data, resulting in the inclusion of 1075 participants after excluding individuals with missing data for sex hormone-related indicators (**Figure 1**).

### 2.2 Definition of exposure

The NHANES database contains serum samples for total testosterone (TT) and estradiol (E2) measured by isotope dilution liquid chromatography-tandem mass spectrometry (ID-LC-MS/MS) at the University of Minnesota. Sex hormone-binding globulin was measured using immuno-188 antibody reactions and chemiluminescence of reaction products. Free testosterone is testosterone that is not bound to albumin and SHBG. The NHANES Laboratory Procedures Manual (LPM) provides comprehensive guidelines for sample collection and handling. Testosterone deficiency was defined as a total testosterone level of less than 300 ng/dL (10). High-sensitivity C-reactive protein measurement is based on a highly sensitive Near Infrared Particle Immunoassay rate method in which anti-CRP antibody-coated particles bind to CRP in the patient sample, resulting in the formation of insoluble aggregates that cause turbidity. Participant serum specimens were transferred to Ottumwa, Iowa for analysis at -30°C. For more specific information on the analyzers and methods used, a document entitled Laboratory Procedures is available on the NHANES Web site.

Based on previous groupings (11), we divided participants into no residual risk group: LDL-C < 2.6 mmol/L, hs-CRP < 3mg/L, residual cholesterol risk only (RCR): LDL-C ≥ 2.6 mmol/L, hs-CRP < 3mg/L, residual inflammation risk only (RIR): LDL-C < 2.6 mmol/L. hs-CRP ≥ 3mg/L, both risk (BR): LDL-C ≥ 2.6 mmol/L, hs-CRP ≥ 3mg/L.

### 2.3 Covariates

Sociodemographic characteristics such as age, education, race/ethnicity, household income, and BMI were collected using the Department of Health and Human Services poverty criteria. Smoking was categorized on the basis of the self-administered questionnaire into three categories: current smoker (smoked moth than 100 cigarettes in life and smoke some days or every day), former smoker (smoked more than 100 cigarettes in life and smoke not at all now), and never smoker (smoked less than 100 cigarettes in life), and alcohol consumption into never, moderate, mild and heavy. We also collected a variety of laboratory data, including uric acid, creatinine, total protein, HDL-C, LDL-C, and total cholesterol. Blood glucose >6.11 mmol/L to 7.1 mmol/L, or oral glucose tolerance test >11.1 mmol/L, or self-reported and physician-diagnosed diabetes was defined as fasting glucose >7.1 mg/dL, or use of insulin was considered a diabetic indicator and self-report taking glucose-lowering medication. Mean blood pressure ≥130 mmHg (systolic) or ≥90 mmHg (diastolic) or use of antihypertensive medication, self-reported and physician-diagnosed hypertension. Participants were taking lipid-lowering drugs, mainly including metabolic agents, antihyperlipidemic agents, hmg-coa reductase inhibitors (statins).

### 2.4 Statistical methods

Each participant’s data was subjected to acceptable statistical analyses, according to NHANES’ complex multistage cluster survey design, which incorporated sample weights and statistical reporting guidelines. For continuous and categorical variables, we reported the mean ± standard deviation (SD) and percentages, respectively. Chi-square tests were employed to detect differences between categorical variables, whereas analysis of variance (ANOVA) was used to analyze continuous data. We examined the independent associations between LDL-C and hs-CRP and sex hormones using multivariate logistic regression, reported as beta values, and risk values between the four groups and testosterone deficiency were expressed as OR. To assess the potential impact of this association, we used a weighted corrected model to adjust for different covariates, with statistically significant differences defined as P < 0.05. We also performed subgroup analysis to identify specific populations. Interaction and stratified analyses were used to evaluate subgroup heterogeneity; P < 0.05 indicates significant interaction term heterogeneity. In addition, we apply the model to investigate whether there is a critical point or not. Based on the model Based on the maximum likelihood value given by the model, the inflection point was determined using a two-step recursive method. Smooth curve fitting and two-segment linear regression modeling were used to determine the association between sex hormones and LDL-C and hs-CRP.

We used R version 4.2.0 (http://www.R-project.org, R Foundation) and EmpowerStats software (www.empowerstats.com; X & Y solutions, Inc., Boston MA) for all analyses.

## 3 Results

1075 people were finally included in the analysis through screening (**Figure 1**). The RIR population was the oldest but the RCR population was the largest, diabetics had a higher risk of inflammation, hypertensives had a higher risk of residual cholesterol, 44.51% of the population was at residual risk even after taking lipid-lowering medications, and had a higher risk of inflammation. The dual-risk group had the highest BMI, protein intake, and uric acid, and the lowest energy intake. For sex hormones, total and free testosterone were low in those at risk for inflammation, sex hormone binding protein was lowest in those at dual risk, and estrogen was low in those at high residual cholesterol risk. The details are in **Table 1**.

**Table 1:** The baseline characteristics of participants, grouped by risk, have been weighted.

	BR	RCR	RIR	Normal	*P-*value
**Age (year)**	51.13 ± 14.31	49.12 ± 15.50	48.79 ± 17.64	48.26 ± 19.01	0.0593
Sex					<0.0001
Male	41.63	53.08	35.80	52.22	
Female	58.37	46.92	64.20	47.78	
Race					0.0265
Mexican American	8.36	7.76	8.58	6.42	
Non-Hispanic White	66.00	69.09	64.79	70.37	
Other Hispanic	7.31	5.79	9.43	5.09	
Non-Hispanic Black	12.21	7.43	12.29	9.27	
Other Race - Including Multi-Racial	6.12	9.94	4.91	8.84	
Poverty					0.5029
<=1	12.38	12.68	13.78	15.38	
>1	87.62	87.32	86.22	84.62	
Education					<0.0001
Less than High school	43.74	44.56	49.61	39.36	
High school	29.35	22.02	21.21	18.76	
More than high school	26.91	33.42	29.18	41.88	
Smoke					0.0124
Never	53.23	54.32	47.08	55.77	
Former	20.12	18.59	13.15	17.20	
Now	26.65	27.09	39.77	27.03	
Alcohol user					0.0916
Never	28.13	28.17	36.92	29.14	
Moderate	15.13	19.54	17.04	17.57	
Mild	39.61	34.33	26.97	37.14	
Heavy	17.13	17.96	19.08	16.15	
Diabetes					<0.0001
No	77.89	87.63	69.87	79.62	
Yes	22.11	12.37	30.13	20.38	
Hypertension					<0.0001
No	50.68	33.15	52.41	34.78	
Yes	49.32	66.85	47.59	65.22	
Take drug					<0.0001
No	31.68	48.50	24.61	41.76	
Yes	68.32	51.50	75.39	58.24	
**BMI (kg/m^2^)**	33.88 ± 7.73	27.67 ± 5.40	33.36 ± 8.17	27.36 ± 6.15	<0.0001
**Total protein (g/L)**	71.47 ± 4.40	71.04 ± 4.34	71.22 ± 4.43	70.37 ± 4.04	0.0010
**Creatinine (µmol/L)**	74.34 ± 34.25	75.28 ± 18.87	73.29 ± 25.23	78.05 ± 39.90	0.1625
**Uric acid (µmol/L)**	343.85 ± 82.68	318.32 ± 75.43	337.28 ± 90.40	308.94 ± 73.04	<0.0001
**TC (mg/dL)**	5.53 ± 0.89	5.55 ± 0.86	4.16 ± 0.70	4.02 ± 0.62	<0.0001
**HDL-C (mg/dL)**	1.33 ± 0.38	1.49 ± 0.44	1.48 ± 0.74	1.55 ± 0.54	<0.0001
**LDL-C (mg/dL)**	3.51 ± 0.74	3.49 ± 0.69	2.10 ± 0.34	2.01 ± 0.38	<0.0001
**Hs-C reactive protein (mg/L)**	8.59 ± 9.13	1.24 ± 0.79	9.61 ± 12.47	1.06 ± 0.78	<0.0001
**Kcal**	1979.28 ± 804.26	2220.03 ± 900.72	2183.32 ± 1015.00	2137.86 ± 840.02	0.0001
**Testosterone (ng/dl)**	182.05 ± 221.68	275.31 ± 276.66	148.53 ± 189.37	262.40 ± 270.56	<0.0001
**Sex hormone binding globulin (nmol/L)**	51.17 ± 33.45	64.07 ± 44.28	62.20 ± 47.63	64.56 ± 43.04	<0.0001
**Estradiol (pg/ml)**	40.10 ± 57.73	33.52 ± 51.30	48.10 ± 54.38	43.69 ± 64.10	0.0023
**Free testosterone (ng/dL)**	3.22 ± 3.84	4.71 ± 4.84	2.52 ± 3.31	4.37 ± 4.77	<0.0001

Data are expressed as weighted proportions for categorical variables (%) and as weighted means ± Standard Error for continuous variables depending on its type.

Grouped by LDL-C and hs-CRP. BR, both risk; RCR, residual cholesterol risk; RIR, residual inflammation risk.

Statistically significant results are bolded.

### 3.1 Relationship of LDL-C and hs-CRP to sex hormones

Within the comprehensively adjusted framework (**Table 2**), an intriguing observation unveiled itself. A significant and inverse correlation emerged between the continuous metric of high-sensitivity C-reactive protein and testosterone (β = -1.98, 95% CI [-3.54, -0.42], p = 0.013), alongside free testosterone (β = -0.04, 95% CI [-0.07, -0.02], p = 0.0002). Furthermore, a nuanced linkage was discerned between the continuous variable LDL-C and sex hormone binding protein (β = -3.61, 95% CI [-5.33, -1.90], p = 0.0003).

**Table 2:** Relationship of LDL-C and hs-CRP to TT, E2, SHBG, and FT.

	Non-adjusted		Adjusted I		Adjusted II	
TT	β	*P*	β	*P*	β	*P*
Hs-CRP	**-4.42 (-6.03, -2.81)**	**<0.0001**	**-3.67 (-5.27, -2.08)**	**<0.0001**	**-1.98 (-3.54, -0.42)**	**0.0130**
T1	Ref				Ref	
T2	**-86.82 (-114.95, -58.69)**	**<0.0001**	**-76.15 (-104.17, -48.12)**	**<0.0001**	**-58.83 (-86.36, -31.30)**	**<0.0001**
T3	**-157.06 (-186.91, -127.22)**	**<0.0001**	**-142.80 (-172.63, -112.98)**	**<0.0001**	**-107.45 (-138.17, -76.73)**	**<0.0001**
LDL-C	-1.09 (-13.84, 11.65)	0.8665	6.75 (-19.25, 5.76)	0.2905	-11.77 (-24.54, 0.99)	0.0710
T1	Ref		Ref		Ref	
T2	-6.41 (-37.70, 24.89)	0.6884	-15.06 (-45.57, 15.46)	0.3337	**-31.38 (-61.37, -1.39)**	**0.0406**
T3	3.83 (-26.37, 34.02)	0.8040	-10.21 (-39.86, 19.44)	0.4999	-26.83 (-57.30, 3.65)	0.0848
SHBG						
Hs-CRP	-0.03 (-0.27, 0.21)	0.8004	-0.21 (-0.43, 0.00)	0.0550	0.02 (-0.20, 0.23)	0.8768
T1	Ref				Ref	
T2	**-4.63 (-8.92, -0.34)**	**0.0346**	**-9.49 (-13.31, -5.67)**	**<0.0001**	**-6.98 (-10.74, -3.22)**	**0.0003**
T3	**-8.84 (-13.41, -4.27)**	**0.0002**	**-13.76 (-17.85, -9.66)**	**<0.0001**	**-8.46 (-12.68, -4.23)**	**<0.0001**
LDL-C	**-3.93 (-5.78, -2.08)**	**<0.0001**	**-2.54 (-4.22, -0.87)**	**0.0030**	**-3.61 (-5.33, -1.90)**	**<0.0001**
T1	Ref				Ref	
T2	**-5.77 (-10.29, -1.25)**	**0.0125**	**-4.24 (-8.32, -0.17)**	**0.0417**	**-7.36 (-11.38, -3.34)**	**0.0003**
T3	**-9.13 (-13.50, -4.77)**	**<0.0001**	**-5.37 (-9.33, -1.41)**	**0.0080**	**-8.52 (-12.60, -4.44)**	**<0.0001**
E2						
Hs-CRP	-0.04 (-0.14, 0.05)	0.3648	-0.03 (-0.13, 0.06)	0.4803	-0.05 (-0.15, 0.05)	0.3018
T1	Ref		Ref		Ref	
T2	-0.92 (-2.57, 0.74)	0.2775	-0.32 (-1.99, 1.34)	0.7027	-0.56 (-2.25, 1.13)	0.5176
T3	0.77 (-0.99, 2.53)	0.3913	1.28 (-0.51, 3.07)	0.1606	0.81 (-1.10, 2.71)	0.4064
LDL-C	-0.06 (-0.77, 0.66)	0.873	-0.15 (-0.87, 0.57)	0.6858	-0.48 (-1.25, 0.29)	0.2241
T1	Ref		Ref		Ref	
T2	-0.26 (-2.00, 1.49)	0.7751	-0.29 (-2.04, 1.45)	0.7421	-0.65 (-2.46, 1.16)	0.4847
T3	-0.29 (-1.98, 1.40)	0.7398	-0.47 (-2.16, 1.23)	0.5877	-1.26 (-3.10, 0.58)	0.1791
FT						
Hs-CRP	**-0.08 (-0.11, -0.06)**	**<0.0001**	**-0.05 (-0.08, -0.03)**	**<0.0001**	**-0.04 (-0.07, -0.02)**	**0.0002**
T1	Ref		Ref		Ref	
T2	**-1.32 (-1.81, -0.82)**	**<0.0001**	**-0.64 (-1.04, -0.25)**	**0.0015**	**-0.58 (-0.98, -0.18)**	**0.0049**
T3	**-2.34 (-2.87, -1.82)**	**<0.0001**	**-1.60 (-2.03, -1.18)**	**<0.0001**	**-1.50 (-1.95, -1.05)**	**<0.0001**
LDL-C	**0.34 (0.12, 0.56)**	**0.0025**	0.07 (-0.11, 0.24)	0.4446	0.04 (-0.15, 0.22)	0.6877
T1	Ref		Ref		Ref	
T2	0.14 (-0.40, 0.68)	0.6070	-0.12 (-0.55, 0.30)	0.5725	-0.23 (-0.67, 0.21)	0.3036
T3	**0.85 (0.33, 1.37)**	**0.0013**	0.19 (-0.22, 0.60)	0.3639	0.07 (-0.37, 0.52)	0.7418

Model I: Age; Race, Education, Poverty.

Model II: Adjust I + BMI; Smoke; Alcohol users; Uric acid; Creatinine; Total protein; Diabetes; Hypertension; Take drugs.

β, effect size for regression; LDL-C, low density lipoprotein cholesterol; hs-CRP, high sensitive c reactive-protein; TT, total testosterone; SHBG, sex hormone binding globulin; E2, estradiol; FT, free testosterone.

Statistically significant results are bolded.

Delving deeper into our investigation, we initiated sensitivity analyses pertaining to the tertile transitions of hs-CRP and LDL-C. This discerning approach revealed noteworthy revelations. Specifically, within the Hs-CRP tertile, the parameters of testosterone (β = -107.45, 95% CI [-138.17, -76.73], p < 0.0001), SHBG (β = -8.46, 95% CI [-5.33, -1.90], p < 0.0001), and free testosterone exhibited diminishment. This phenomenon was mirrored within the LDL-C tertile, where testosterone (β = -1.50, 95% CI [-1.95, -1.05], p < 0.0001) bore the most pronounced decrease. Intriguingly, testosterone displayed a notable decrease within the dichotomous tertile of LDL-C (β = -31.38, 95% CI [-61.37, -1.39], p = 0.0406), whereas SHBG registered a similar pattern within the tertiary tertile (β = -8.52, 95% CI [-12.60, -4.44], p < 0.0001).

Additionally, an insightful exploration into the realms of high residual cholesterol risk and high inflammation risk groups unfolded (**Table 3**). The outcomes proved captivating; testosterone exhibited a substantial decline within the high residual cholesterol risk and high inflammation risk groups (β = -79.37, 95% CI [-112.74, -46.00], p < 0.0001), alongside a marked decrease within the high inflammation group (β = 66.50, 95% CI [-107.92, -25.09], p < 0.0001). Amidst this landscape, SHBG displayed a parallel descent, registering significant reductions within both the BR (β = -8.29, 95% CI [-12.86, -3.72], p = 0.0004) and RCR (β = -5.48, 95% CI [-9.35, -1.62], p = 0.0055) populations. Conversely, estrogen remained devoid of significant alterations.

**Table 3:** Relationship between TT, E2, SHBG and FT in different risk subgroups.

Exposure	β	P
TT		
Normal	Ref	
RCR	-12.23 (-40.58, 16.13)	0.3982
RIR	**-66.50 (-107.92, -25.09)**	**0.0017**
BR	**-79.37 (-112.74, -46.00)**	**<0.0001**
SHBG		
Normal	Ref	
RCR	**-5.48 (-9.35, -1.62)**	**0.0055**
RIR	-4.47 (-10.18, 1.24)	0.1252
BR	**-8.29 (-12.86, -3.72)**	**0.0004**
E2		
Normal	Ref	
RCR	-0.86 (-2.60, 0.87)	0.3300
RIR	1.28 (-1.29, 3.84)	0.3287
BR	0.38 (-1.67, 2.44)	0.7141
FT		
Normal	Ref	
RCR	0.22 (-0.20, 0.63)	0.3056
RIR	**-1.00 (-1.61, -0.40)**	**0.0012**
BR	**-0.97 (-1.46, -0.48)**	**0.0001**

β, effect size for regression; TT, total testosterone; SHBG, sex hormone binding globulin; E2, estradiol; FT, free testosterone; BR, both risk; RCR, residual cholesterol risk; RIR, residual inflammation risk; SHBG, sex hormone binding globulin.

Statistically significant results are bolded.

**Figure 2:**
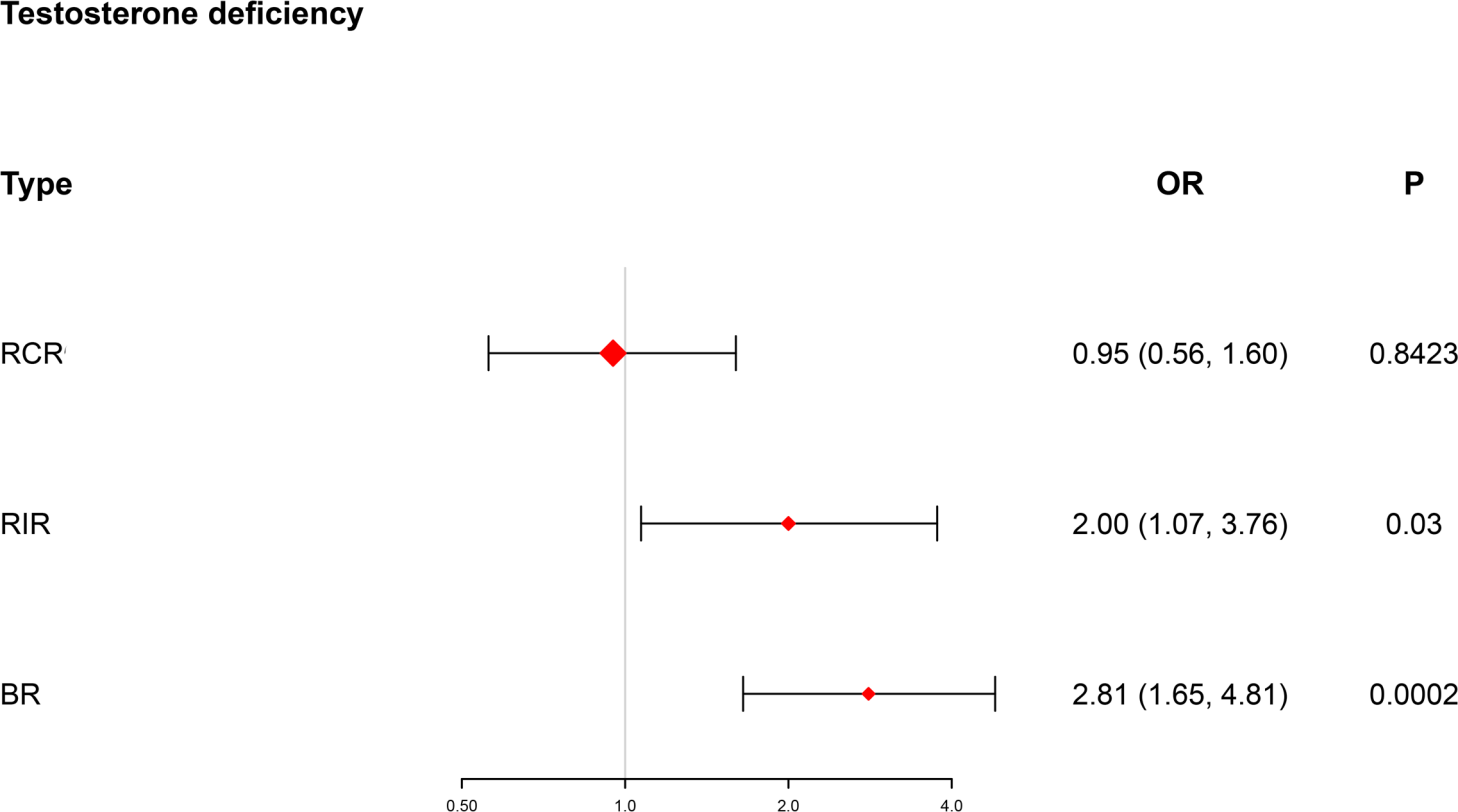
Forest plot showing testosterone deficiency in different risk subgroups. BR, both risk; RCR, residual cholesterol risk; RIR, residual inflammation risk; OR, odds ratio (logarithmic scale); CI, confidence interval.

A pivotal synthesis of our discoveries unveiled substantial risk disparities among men grappling with testosterone deficiency (**Figure 2**). Specifically, within the BR context, these individuals were exposed to a formidable 1.81-fold higher risk (OR = 2.81, 95% CI [1.65, 4.81], p = 0.0002), and within the RIR landscape, a comparable 1-fold elevated risk (OR = 2.00, 95% CI [1.07, 3.76], p = 0.03) came to light when juxtaposed with the normal population.

### 3.2 LDL-C and hs-CRP dose response and thresholds to sex hormones

**Figure 3:**
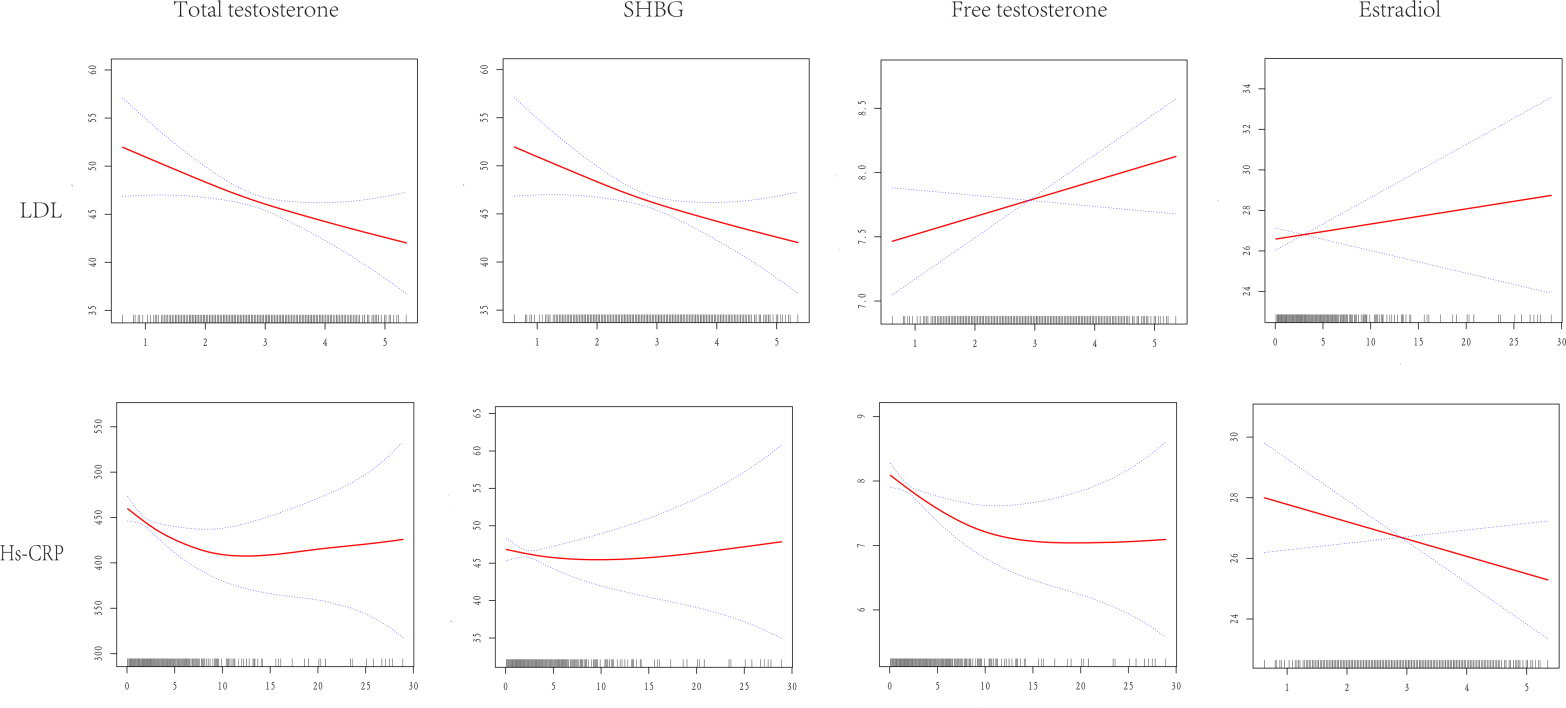
Dose-response relationship between LDL-C and hs-CRP and TT, SHBG, E2, FT. Adjustments were made for all covariates except effect modifiers. LDL-C, low density lipoprotein cholesterol; hs-CRP, high sensitive c reactive-protein; TT, total testosterone; SHBG, sex hormone binding globulin; E2, estradiol; FT, free testosterone.

The amalgamation of generalized linear models and the finesse of smoothed curve fitting was harnessed to orchestrate a synthesis of the intricate interplay between sex hormones and LDL-C as well as hs-CRP, as elegantly illustrated in **Figure 3** and meticulously detailed in **Table 4**.

**Table 4:** Two-way linear regression and log-likelihood ratio tests explained the analysis of threshold effects of LDL-C and hs-CRP on TT, SHBG, E2, and FT.

	ULR Test β (95% CI)	PLR Test β (95% CI)	LRT test P-value
LDL-C			
Total testosterone			
<1.89	0.42 (-11.81, 12.65)	-68.84 (-143.97, 6.30)	0.063
≥1.89		6.94 (-7.13, 21.01)	
SHBG			
<2.46	-1.91 (-3.64, -0.18)	-5.79 (-10.95, -0.63)	0.112
≥2.46		-0.49 (-2.97, 2.00)	
Estradiol			
<1.58	-0.53 (-1.32, 0.25)	2.46 (-5.82, 10.74)	0.47
≥1.58		-0.64 (-1.48, 0.20)	
Free testosterone			
<1.89	0.14 (-0.04, 0.32)	-0.72 (-1.83, 0.39)	0.118
≥1.89		0.22 (0.02, 0.43)	
Hs-CRP			
Total testosterone			
<1	-2.80 (-5.67, 0.07)	-69.51 (-111.71, -27.31)	**0.002**
≥1		-1.54 (-4.50, 1.42)	
SHBG			
<0.8	-0.03 (-0.43, 0.38)	-12.77 (-20.69, -4.86)	**0.001**
≥0.8		0.12 (-0.29, 0.54)	
Estradiol			
<0.3	-0.07 (-0.11, 0.26)	-12.90 (-28.20, 2.41)	0.091
≥0.3		0.08 (-0.10, 0.27)	
Free testosterone			
<9.2	-0.06 (-0.10, -0.01)	-0.13 (-0.20, -0.05)	**0.022**
≥9.2		0.03 (-0.06, 0.11)	

ULR, univariate linear regression; PLR, piecewise linear regression; LRT, logarithmic likelihood ratio test, statistically significant: p < 0.05. TT, total testosterone; SHBG, sex hormone binding globulin; E2, estradiol; FT, free testosterone.

Statistically significant results are bolded.

Exemplifying the results of this endeavor, we divulge that LDL-C unfolded its linear connectivity with TT, SHBG, E2, and FT underpinned by p-values that surpassed the threshold of significance at > 0.05. Conversely, a discernibly more complex duality took shape within the interface of HS-CRP and total testosterone. As per the comprehensive bipartite linear regression model, our computation revealed an inflection point at 1. Precisely, at the <1 stratum, the estimation loomed at -69.51 (95% CI: -111.71, -27.31), while at the ≥1 stratum, it settled at -1.54 (95% CI: -4.50, 1.42). This phenomenon was endorsed by the log-likelihood ratio test with a consequential P-value of 0.002. The narrative of SHBG, within its own inflection point at 0.8, unfolded as follows: at <0.8, a pronounced descent of -12.77 (95% CI: -20.69, -4.86) emerged, and at ≥0.8, a gentle ascent of 0.12 (95% CI: -0.29, 0.54) came to the fore, with a commanding P-value of 0.001. Echoing a similar pattern, the narrative of free testosterone, marked by its inflection point at 9.2, illustrated a notable drop at <9.2, quantified at -0.13 (95% CI: -0.20, -0.05), and a muted increase at ≥9.2, tabulated at 0.03 (95% CI: -0.06, 0.11), although its significance remained intact with P-values ≥ 0.002. Not to be overlooked, the relationship between HS-CRP and estradiol presented a linear association, albeit with a p-value of 0.091, evoking a sense of subtlety in the overall context.

### 3.3 Subgroup analysis

The homeostatic interactions among sex hormones, hs-CRP, and LDL-C were carefully analyzed by subgroup analyses, which revealed some intriguing findings. Only hs-CRP and estradiol were significantly different in the interaction test in diabetic patients (P= 0.0204), indicating that the higher the degree of inflammation, the higher the estradiol in diabetic patients. The other subgroups analyzed did not show significant heterogeneity in the results of the interaction effect test, suggesting that there were no significant differences in the associations between the different subgroups and that the results were stable (**Table 5**).

**Table 5:** Subgroup analysis.

	LDL-C			Hs-CRP		
Testosterone	β (95% CI)	P	P for interaction	β (95% CI)	P	P for interaction
Take drugs			0.1058			0.4029
No	-5.50 (-22.29, 11.30)	0.5213		-3.72 (-8.11, 0.68)	0.0979	
Yes	4.68 (-12.84, 22.21)	0.6007		-2.53 (-5.88, 0.81)	0.1387	
Diabetes			0.4079			0.0755
No	4.80 (-8.83, 18.43)	0.4905		-4.70 (-7.95, -1.46)	0.0046	
Yes	-11.20 (-35.57, 13.16)	0.3686		0.67 (-4.00, 5.34)	0.7781	
Hypertension			0.81			0.2054
No	-0.11 (-19.30, 19.09)	0.9913		-1.79 (-5.70, 2.12)	0.3711	
Yes	4.43 (-10.31, 19.17)	0.5563		-4.69 (-8.45, -0.93)	0.0148	
SHBG						
Take drugs			0.4013			0.7519
No	-2.82 (-4.88, -0.76)	0.0075		-1.29 (-4.05, 1.47)	0.3614	
Yes	-0.15 (-0.70, 0.39)	0.5778		-0.03 (-0.56, 0.50)	0.9022	
Diabetes			0.3085			0.5962
No	-1.70 (-3.52, 0.11)	0.0661		-0.12 (-0.55, 0.32)	0.605	
Yes	**-4.28 (-7.99, -0.57)**	**0.025**		0.08 (-0.63, 0.78)	0.8343	
Hypertension			0.9059			0.9772
No	-2.21 (-5.16, 0.73)	0.1416		-0.07 (-0.67, 0.52)	0.8089	
Yes	-1.38 (-3.29, 0.53)	0.158		-0.03 (-0.52, 0.46)	0.8999	
Estradiol						
Take drugs			0.505			0.542
No	-0.48 (-1.49, 0.53)	0.3497		-0.49 (-1.67, 0.69)	0.4128	
Yes	-0.15 (-0.41, 0.12)	0.2725		0.10 (-0.13, 0.32)	0.4131	
Diabetes			0.9209			**0.0204**
No	-0.35 (-1.16, 0.47)	0.4087		-0.15 (-0.35, 0.05)	0.1318	
Yes	-0.68 (-2.52, 1.16)	0.4715		**0.39 (0.03, 0.74)**	**0.0328**	
Hypertension			0.4572			0.1703
No	-0.02 (-1.23, 1.19)	0.977		0.16 (-0.09, 0.41)	0.2003	
Yes	-0.71 (-1.65, 0.24)	0.1427		-0.18 (-0.42, 0.06)	0.1427	
Free testosterone						
Take drugs			0.4874			0.395
No	0.18 (-0.10, 0.46)	0.2179		0.18 (-0.08, 0.44)	0.1685	
Yes	**-0.07 (-0.15, -0.00)**	**0.0493**		-0.05 (-0.10, 0.00)	0.0521	
Diabetes			0.7392			0.1395
No	**0.23 (0.01, 0.45)**	**0.0428**		-0.08 (-0.13, -0.03)	0.0033	
Yes	0.11 (-0.22, 0.45)	0.5119		-0.01 (-0.07, 0.05)	0.7627	
Hypertension			0.7343			0.1744
No	0.13 (-0.12, 0.39)	0.3105		-0.03 (-0.08, 0.02)	0.2891	
Yes	0.20 (-0.06, 0.45)	0.1326		**-0.09 (-0.16, -0.03)**	**0.0054**	

β, effect size; CI, confidence interval; LDL-C, low density lipoprotein cholesterol; hs-CRP, high sensitive c reactive-protein; TT, total testosterone; SHBG, sex hormone binding globulin; E2, estradiol; FT, free testosterone. All relevant variables were adjusted except for those in the stratified analysis (Model 3). Statistically significant results are bolded.

Within this nuanced exploration, a distinctive pattern emerged wherein the relationship between SHBG and elevated LDL-C displayed a more marked decline, notably evident among individuals who were not utilizing lipid-lowering medications (β = -2.82, 95% CI [-4.88, -0.76], p = 0.0075). Parallel to this, a discernible distinction was observed within non-diabetic patients, where hs-CRP exhibited a statistically significant inverse correlation with both total testosterone (β = -4.70, 95% CI [-7.95, -1.46], p = 0.0046) and free testosterone (β = -0.08, 95% CI [-0.13, -0.03], p = 0.0033). Additionally, a positive correlation surfaced between LDL-C and free testosterone (β = -0.23, 95% CI [0.01, 0.45], p = 0.0428) within this same subgroup. The narrative took a different turn for diabetic patients, where LDL-C displayed an adverse relationship with SHBG (β = -4.28, 95% CI [-7.99, -0.57], p = 0.025). As the analysis pivoted towards the realm of hypertension, a notable constancy was witnessed in the relationship between LDL-C and each respective indicator. Yet, a distinctive dynamic emerged in the context of hypertensive patients, where hs-CRP assumed significance by evincing a substantial inverse correlation with both total testosterone (β = -4.69, 95% CI [-8.45, -0.93], p = 0.0148) and free testosterone (β = -0.09, 95% CI [-0.16, -0.03], p = 0.0054).

## 4 Discussion

Both LDL-C and hs-CRP exhibit robust connections with sex hormone dynamics. Noteworthy alterations in sex hormone concentrations surfaced in individuals confronting cholesterol-driven residual risk and inflammation-associated residual risk. However, within the realm of adult male physiology, inflammation emerged as a more potent influencer of testosterone levels compared to LDL cholesterol. Upon meticulous adjustment and refinement of the model, LDL cholesterol relinquished its autonomous correlation with testosterone, while hs-CRP retained its significant, independent and adverse correlation with both testosterone and free testosterone. The amalgamation of these factors posed a heightened risk for testosterone insufficiency. Furthermore, an autonomous and unfavorable link between LDL-C and sex hormone binding globulin (SHBG) was distinctly discerned. In the intricate tapestry of male hormonal milieu, estrogen did not manifest any significant associations.

Nevertheless, within the context of subgroup analysis, the presence or absence of lipid-lowering medications exerted a discernible influence solely on the interplay between SHBG and free testosterone, along with LDL-C. All other interrelations retained their unaltered constancy. On a divergent note, among diabetic patients, the responsiveness of both risks to the sway of sex hormones seemed comparatively muted when juxtaposed with normative patients. In the domain of hypertensive patients, the interplay of high inflammation and sex hormones yielded a heightened sensitivity, particularly in relation to testosterone and free testosterone. This phenomenon illuminates the elevated vulnerability of male participants situated within the high-inflammation cohort to testosterone deficiency. Moreover, while the presence of high residual cholesterol in isolation did not distinctly manifest a specific risk profile, participants ensnared in the dual-risk realm exhibited an escalated susceptibility to testosterone deficiency in comparison to their single-risk counterparts.

The heightened state of inflammation is likely an outcome of interleukin-1 (IL-1), interleukin-6 (IL-6), interleukin-17 (IL-17), and tumor necrosis factor, which collectively perturb the equilibrium of the hypothalamic-pituitary-gonadal axis, consequently leading to a decrement in testosterone synthesis (12), Further, evidence has indicated that individuals burdened with obesity, due to augmented visceral adiposity, undergo a conversion of testosterone to estrogen within inflamed adipose tissue, thereby contributing to a depletion of testosterone levels within the organism (13, 14). In preclinical studies, androgens have demonstrated anti-inflammatory attributes with regard to inflammation markers, while conversely, inflammation can impede the production of testosterone (15, 16) Investigations have also unveiled an inverse association between the sex hormone testosterone and C-reactive protein (CRP) concentration, as well as a similar inverse correlation between estradiol and CRP. Strikingly, SHBG is independently correlated with white blood cell count, irrespective of CRP, thereby suggesting that both excessive androgen and inadequate estrogen levels can elicit anti-inflammatory effects within the male populace (17). The repercussions of excessive inflammation on cellular functioning are notably profound. The implementation of anti-inflammatory interventions can enhance the abundance of inflammation-sensitive T-cells, thereby affording relief from testosterone insufficiency. Furthermore, empirical data has spotlighted that certain dietary choices exhibit pro-inflammatory tendencies, which, when consumed excessively, can indeed impact sex hormone equilibrium, particularly testosterone. Hence, an awareness of the imperative to adopt a low pro-inflammatory diet is incumbent (18). Sex hormone binding globulin, a hepatically produced protein, aligns itself with levels of both high-density lipoprotein cholesterol (HDL-C) and low-density lipoprotein cholesterol (LDL-C). The principal avenue through which testosterone exerts its influence is facilitated by the conveyance of SHBG within the serum. Diminished free testosterone precipitates an elevation in SHBG, thereby fostering the liberation of luteinizing hormone, which in turn augments testosterone synthesis. A discernible negative correlation between SHBG and triglycerides (TG) and LDL-C, coupled with a positive correlation with HDL-C, substantiates this phenomenon2. The nexus between adipose accumulation and insulin resistance, intricately intertwined with fat metabolism, may underpin the association with reduced SHBG levels (19, 20). Notably, a previous inquiry demonstrated a lack of link between SHBG and LDL-C in males, a variance that might be attributed to dissimilarities in ethnicity and test methodologies. Conversely, SHBG exhibits a robust correlation with HDL-C and serves as a pivotal protein in the orchestration of lipid regulation (21). In normoglycemic men, LDL was independently and significantly linked to both SHBG and total testosterone, with this relationship intensifying in the nonobese subset (22).

Symptoms associated with testosterone deficiency (TD) can exhibit variability contingent upon the extent of the deficiency and the timing of its onset. These manifestations may encompass diminished libido and erectile dysfunction, dwindled muscle mass and strength, fatigue and depleted vitality, alterations in mood accompanied by depression, reduced bone density, and even weight loss. Numerous models have been developed predicated on rudimentary metrics, ostensibly poised to prognosticate the advent of TD, such as the visceral adiposity index (VAI) and the triglyceride-to-HDL cholesterol ratio (TG/HDL-C), yet the prognostic potency of these models remains somewhat insubstantial (23). Metrics such as TyG, LAP, VAI, and HOMA-IR have emerged as adept predictors of TD, characterized by commendable specificity and sensitivity across both diabetic and non-diabetic cohorts (24, 25). Notably, within the realm of participants categorized under the RCR cohort, the significance of TD waned due to the pronounced gravity of the deficiency itself. However, in the RIR and BR cohorts, the risk of TD experienced a marked elevation. This underscores the necessity for individuals, particularly those who are non-diabetic and hypertensive, afflicted by high levels of inflammation, to exercise vigilance towards potential fluctuations in testosterone levels, given the amplified risk entailed in such circumstances.

Nonetheless, our study remains vulnerable to certain limitations which merit scrupulous acknowledgment: 1. There are limitations in the sample size for inclusion; 2. Cross-sectional studies are limited by the simplification of biomarkers, population heterogeneity, and lack of causal evidence, and should be cautious about generalizing to populations with different genetic backgrounds or environments, and future integration of multi-omic data and cross-ethnic validation are needed to improve the models. Subgroups based on LDL-C and hs-CRP thresholds may not be directly translated into actionable clinical insights; 3. Results from subgroup analyses need to be further explored through multi-omics integration (e.g., lipoprotein subfractions vs. tissue-specific inflammatory markers) and cross-ethnic validation, and hypothesis-generating attributes of the subgroup results should be interpreted with caution to avoid over-interpretation as a basis for clinical decision-making. Initially, our data elucidating the adverse interplay between RCR and SHBG warrants prudent scrutiny. Though the pivotal role of HDL-C in SHBG dynamics is firmly established, the extent to which SHBG is diminished in individuals confronting high residual cholesterol risk has not garnered widespread empirical substantiation. Moreover, while it is undeniable that patients with RCR did indeed exhibit diminished testosterone levels, it is imperative to underscore that while a direct correlation eluded our observation, the revelation of such an association warrants the conduction of an extensive array of investigations for future affirmation. Secondary to this, while our findings allude to the robust concordance between heightened inflammation and testosterone levels, and the linkage between high residual cholesterol risk and SHBG, with potential utility in prognosticating sex hormone perturbations, the inherent constraints of cross-sectional studies preclude us from delineating the underlying mechanistic underpinnings. This necessitates the undertaking of more comprehensive investigations to uncover the intricate mechanisms at play. Furthermore, it is worth noting that our classification of RCR was based upon LDL-C levels. Nonetheless, there remains an intriguing query as to whether other lipid constituents contribute to residual risk, such as triglycerides and the cholesterol content within triglyceride-rich lipoproteins. Lastly, our study was predicated upon the expansive NHANES database, encompassing the demography of the U.S. populace and its ethnic diversity. However, it is incumbent upon us to acknowledge that the generalizability of our findings to other ethnicities and races may be subject to limitation.

## Data Availability

The datasets presented in this study can be found in online repositories. The names of the repository/repositories and accession number(s) can be found below: https://wwwn.cdc.gov/nchs/nhanes.
