# Supplementary material for: Impact of dual residual risk of cholesterol and inflammation on adult male sex hormones: a cross-sectional study from NHANES
Source: Front Endocrinol (Lausanne). 2025 Mar 10;16:1526056. doi: 10.3389/fendo.2025.1526056 (PMC11931246; doi:10.3389/fendo.2025.1526056)
Supplement: Supplementary file 1 [file Table1.docx]

**Appendix 1**

Classification of patients according to testosterone deficiency

|  | >=300 | <300 | P value |
| --- | --- | --- | --- |
| **Age (year)** | 47.97 ± 16.57 | 51.97 ± 15.43 | 0.0041 |
| **Sex** |  |  | <0.0001 |
| Male | 99.60 | 15.49 |  |
| **Race** |  |  | 0.5408 |
| Mexican American | 8.50 | 7.50 |  |
| Non-Hispanic White | 67.10 | 70.70 |  |
| Other Hispanic | 7.56 | 4.69 |  |
| Non-Hispanic Black | 8.12 | 10.04 |  |
| Other Race - Including Multi-Racial | 8.73 | 7.08 |  |
| **Poverty** |  |  | 0.4194 |
| <=1 | 11.38 | 13.60 |  |
| >1 | 88.62 | 86.40 |  |
| **Education** |  |  | 0.3628 |
| Less than High school | 42.39 | 44.11 |  |
| High school | 22.84 | 26.45 |  |
| More than high school | 34.77 | 29.44 |  |
| **Smoke** |  |  | <0.0001 |
| Never | 51.35 | 37.87 |  |
| Former | 22.17 | 10.47 |  |
| Now | 26.48 | 51.66 |  |
| **Alcohol user** |  |  | 0.0008 |
| Never | 21.75 | 34.45 |  |
| Moderate | 13.69 | 6.55 |  |
| Mild | 41.85 | 41.45 |  |
| Heavy | 22.70 | 17.55 |  |
| **Diabetes** |  |  | <0.0001 |
| No | 84.34 | 62.52 |  |
| Yes | 15.66 | 37.48 |  |
| **Hypertension** |  |  | 0.0003 |
| No | 39.17 | 54.52 |  |
| Yes | 60.83 | 45.48 |  |
| **Type** |  |  | 0.0002 |
| RCRO | 42.51 | 31.42 |  |
| BR | 18.73 | 31.41 |  |
| RIR | 7.52 | 12.17 |  |
| Normal | 31.24 | 25.00 |  |
| **Take drug** |  |  | <0.0001 |
| No | 50.35 | 29.28 |  |
| Yes | 49.65 | 70.72 |  |
| **BMI (kg/m^2^)** | 28.57 ± 6.01 | 34.55 ± 7.05 | <0.0001 |
| **Total protein (g/L)** | 71.62 ± 4.18 | 71.19 ± 4.13 | 0.2247 |
| **Creatinine (µmol/L)** | 84.65 ± 32.83 | 85.90 ± 23.29 | 0.6376 |
| **Uric acid (µmol/L)** | 352.82 ± 70.29 | 392.48 ± 73.03 | <0.0001 |
| **TC (mg/dL)** | 4.85 ± 1.07 | 4.81 ± 1.03 | 0.6591 |
| **HDL-C (mg/dL)** | 1.33 ± 0.39 | 1.14 ± 0.31 | <0.0001 |
| **LDL-C (mg/dL)** | 2.95 ± 0.96 | 2.90 ± 0.86 | 0.5338 |
| **Kcal** | 2465.71 ± 937.95 | 2299.50 ± 874.06 | 0.0352 |
| **Hs-C reactive protein (mg/L)** | 2.91 ± 5.63 | 5.06 ± 7.46 | <0.0001 |

Data are expressed as weighted proportions for categorical variables and as weighted means ± Standard Error for continuous variables depending on its type.

Grouped by LDL-C and hs-CRP. BR, both risk; RCR, residual cholesterol risk; RIR, residual inflammation risk

**Appendix 2.1**

Results of the multiple regression analysis for female

|  | Non-adjusted |  | Adjusted Ⅰ |  | Adjusted Ⅱ |  |
| --- | --- | --- | --- | --- | --- | --- |
| **TT** | β | *P* | β | *P* | β | *P* |
| Hs-CRP | -0.13 (-0.27, 0.01) | 0.0697 | -0.12 (-0.26, 0.03) | 0.1104 | -0.09 (-0.24, 0.06) | 0.2238 |
| T1 | Ref |  |  |  | Ref |  |
| T2 | -3.29 (-7.19, 0.61) | 0.0987 | -3.13 (-7.17, 0.91) | 0.1289 | **-3.81 (-7.88, 0.27)** | **0.0675** |
| T3 | **-5.81 (-9.53, -2.09)** | **0.0023** | **-5.78 (-9.68, -1.88)** | **0.0038** | **-6.24 (-10.32, -2.15)** | **0.0028** |
| LDL-C | **1.74 (0.20, 3.28)** | **0.0275** | **2.02 (0.47, 3.58)** | **0.0110** | **1.74 (0.09, 3.38)** | **0.0389** |
| T1 | Ref |  | Ref |  | Ref |  |
| T2 | -0.48 (-4.10, 3.13) | 0.7927 | -0.34 (-3.97, 3.29) 0.8537 | 0.3337 | -0.74 (-4.46, 2.97) | 0.6957 |
| T3 | 3.07 (-0.47, 6.61) | 0.0892 | 3.78 (0.20, 7.36) 0.0387 | 0.4999 | 2.90 (-0.90, 6.69) | 0.1349 |
| **SHBG** |  |  |  |  |  |  |
| Hs-CRP | **-0.53 (-0.78, -0.28)** | **<0.0001** | **-0.51 (-0.75, -0.26)** | **<0.0001** | **-0.33 (-0.58, -0.09)** | **0.0076** |
| T1 | Ref |  |  |  | Ref |  |
| T2 | **-9.02 (-15.83, -2.20)** | **0.0097** | **-8.92 (-15.93, -1.90)** | **0.0129** | -6.01 (-12.82, 0.79) | 0.0836 |
| T3 | **-23.14 (-29.63, -16.65)** | **<0.0001** | **-22.93 (-29.70, -16.17)** | **<0.0001** | **-15.42 (-22.23, -8.60)** | **<0.0001** |
| LDL-C | -2.79 (-5.59, 0.00) | 0.0504 | **-3.38 (-6.19, -0.58)** | **0.0182** | **-2.91 (-5.73, -0.10)** | **0.0429** |
| T1 | Ref |  |  |  | Ref |  |
| T2 | -4.83 (-11.32, 1.66) | 0.1450 | -4.79 (-11.25, 1.67) | 0.1466 | -5.71 (-11.99, 0.57) | 0.0749 |
| T3 | **-7.58 (-13.93, -1.24)** | **0.0194** | **-8.49 (-14.85, -2.12)** | **0.0091** | **-8.79 (-15.18, -2.40)** | **0.0072** |
| **E2** |  |  |  |  |  |  |
| Hs-CRP | -0.27 (-0.75, 0.20) | 0.2611 | -0.23 (-0.66, 0.20) | 0.2977 | -0.22 (-0.66, 0.23) | 0.3432 |
| T1 | Ref |  | Ref |  | Ref |  |
| T2 | **-23.59 (-36.86, -10.31)** | **0.0005** | -12.31 (-24.62, -0.00) | 0.0503 | **-12.65 (-25.16, -0.14)** | **0.0477** |
| T3 | **-21.12 (-33.79, -8.45)** | **0.0011** | -10.89 (-22.77, 1.00) | 0.0730 | -9.78 (-22.31, 2.75) | 0.1263 |
| LDL-C | **-8.55 (-13.79, -3.31)** | **0.0014** | -3.45 (-8.19, 1.29) | 0.1545 | -4.25 (-9.29, 0.80) | 0.0992 |
| T1 | Ref |  | Ref |  | Ref |  |
| T2 | -2.20 (-14.47, 10.08) | 0.7257 | 3.05 (-7.99, 14.10) | 0.5882 | 2.74 (-8.62, 14.11) | 0.6362 |
| T3 | **-19.80 (-31.81, -7.78)** | **0.0013** | -7.36 (-18.25, 3.54) | 0.1860 | -9.07 (-20.68, 2.54) | 0.1260 |
| **FT** |  |  |  |  |  |  |
| Hs-CRP | 0.00 (-0.00, 0.00) | 0.7590 | 0.00 (-0.00, 0.00) | 0.6883 | -0.00 (-0.00, 0.00) | 0.9723 |
| T1 | Ref |  | Ref |  | Ref |  |
| T2 | -0.03 (-0.07, 0.02) | 0.2703 | -0.02 (-0.06, 0.03) | 0.4330 | -0.04 (-0.08, 0.01) | 0.1229 |
| T3 | 0.01 (-0.04, 0.05) | 0.7361 | 0.01 (-0.03, 0.06) | 0.5753 | -0.02 (-0.06, 0.03) | 0.42061 |
| LDL-C | **0.02 (0.00, 0.04)** | **0.0173** | **0.03 (0.01, 0.04)** | **0.0026** | **0.02 (0.00, 0.04)** | **0.0215** |
| T1 | Ref |  | Ref |  | Ref |  |
| T2 | 0.01 (-0.03, 0.05) | 0.5204 | 0.02 (-0.03, 0.06) | 0.4530 | 0.01 (-0.03, 0.05) | 0.5294 |
| T3 | **0.04 (0.00, 0.08)** | **0.0351** | **0.06 (0.02, 0.10)** | **0.0059** | **0.05 (0.00, 0.09)** | **0.0296** |

Model I: Age; Race, Education, Poverty.

Model Ⅱ: Adjust I + BMI; Smoke; Alcohol users; Uric acid; Creatinine; Total protein; Diabetes; Hypertension; Take drugs.

β, effect size for regression; LDL-C, low density lipoprotein cholesterol; hs-CRP, high sensitive c reactive-protein; TT, total testosterone; SHBG, sex hormone binding globulin; E2, estradiol; FT, free testosterone.

**Appendix 2.1**

Regression equations for different risk groupings

| Exposure | sex= Male | sex= Female | Total |
| --- | --- | --- | --- |
| TT |  |  |  |
| Normal | Ref | Ref | Ref |
| RCR | -12.23 (-40.58, 16.13) 0.3982 | 3.20 (-0.79, 7.20) 0.1167 | -2.56 (-17.62, 12.51) 0.7395 |
| RIR | -66.50 (-107.92, -25.09) 0.0017 | -2.75 (-7.67, 2.18) 0.2749 | -33.48 (-53.53, -13.43) 0.0011 |
| BR | -79.37 (-112.74, -46.00) <0.0001 | -1.48 (-5.70, 2.74) 0.4926 | -35.38 (-52.14, -18.62) <0.0001 |
| SHBG |  |  |  |
| Normal | Ref | Ref | Ref |
| RCRO | -5.48 (-9.35, -1.62) 0.0055 | -2.18 (-8.86, 4.50) 0.5224 | -4.54 (-8.37, -0.71) 0.0204 |
| RIR | -4.47 (-10.18, 1.24) 0.1252 | -7.60 (-15.87, 0.67) 0.0720 | -6.19 (-11.34, -1.03) 0.0187 |
| BR | -8.29 (-12.86, -3.72) 0.0004 | -15.21 (-22.23, -8.19) <0.0001 | -13.11 (-17.38, -8.85) <0.0001 |
| Estradiol |  |  |  |
| Normal | Ref | Ref | Ref |
| RCRO | -0.86 (-2.60, 0.87) 0.3300 | -9.80 (-19.39, -0.22) 0.0453 | -7.68 (-12.75, -2.61) 0.0031 |
| RIR | 1.28 (-1.29, 3.84) 0.3287 | -3.41 (-15.21, 8.38) 0.5709 | 1.77 (-5.02, 8.57) 0.6086 |
| BR | 0.38 (-1.67, 2.44) 0.7141 | -8.16 (-18.29, 1.97) 0.1148 | -5.71 (-11.38, -0.04) 0.0485 |
| Free testosterone |  |  |  |
| Normal | Ref | Ref | Ref |
| RCRO | 0.22 (-0.20, 0.63) 0.3056 | 0.03 (-0.01, 0.08) 0.1244 | 0.28 (0.04, 0.52) 0.0216 |
| RIR | -1.00 (-1.61, -0.40) 0.0012 | -0.00 (-0.06, 0.05) 0.9935 | -0.54 (-0.86, -0.22) 0.0010 |
| BR | -0.97 (-1.46, -0.48) 0.0001 | 0.04 (-0.01, 0.08) 0.1209 | -0.30 (-0.57, -0.03) 0.0273 |

Model I: Age; Race, Education, Poverty.

Model Ⅱ: Adjust I + BMI; Smoke; Alcohol users; Uric acid; Creatinine; Total protein; Diabetes; Hypertension; Take drugs.

β, effect size for regression; LDL-C, low density lipoprotein cholesterol; hs-CRP, high sensitive c reactive-protein; TT, total testosterone; SHBG, sex hormone binding globulin; E2, estradiol; FT, free testosterone.

**Appendix 3**

Female subgroup analysis and interaction test P for interaction

|  | LDL-C |  |  | hs-CRP |  |  |
| --- | --- | --- | --- | --- | --- | --- |
|  | β (95% CI) | P | P for interaction | β (95% CI) | P | P for interaction |
| Testosterone |  |  |  |  |  |  |
| Take drugs |  |  | 0.70240 |  |  | 0.21820 |
| No | 2.76 (-0.17, 5.69) | 0.06540 |  | -0.65 (-1.32, 0.03) | 0.06030 |  |
| Yes | 1.97 (0.14, 3.81) | 0.03580 |  | -0.27 (-0.61, 0.08) | 0.12760 |  |
| Diabetes |  |  | 0.75760 |  |  | 0.93980 |
| No | 2.29 (0.34, 4.24) | 0.02190 |  | -0.37 (-0.77, 0.03) | 0.06750 |  |
| Yes | 1.94 (-0.57, 4.45) | 0.13100 |  | -0.34 (-0.80, 0.11) | 0.14210 |  |
| Hypertension |  |  | 0.80090 |  |  | 0.25940 |
| No | 2.54 (0.07, 5.02) | 0.04430 |  | -0.16 (-0.47, 0.16) | 0.32430 |  |
| Yes | 2.10 (-0.37, 4.58) | 0.09620 |  | -0.67 (-1.21, -0.13) | 0.01460 |  |
| SHBG |  |  |  |  |  |  |
| Take drugs |  |  | 0.27860 |  |  | 0.15890 |
| No | -0.67 (-4.52, 3.18) | 0.73290 |  | -0.44 (-1.31, 0.44) | 0.33190 |  |
| Yes | -3.87 (-7.65, -0.09) | 0.04510 |  | 0.15 (-0.57, 0.87) | 0.68580 |  |
| Diabetes |  |  | 0.15330 |  |  | 0.26830 |
| No | -0.55 (-3.65, 2.56) | 0.73100 |  | -0.16 (-0.79, 0.48) | 0.62870 |  |
| Yes | -4.86 (-10.39, 0.68) | 0.08720 |  | 0.34 (-0.73, 1.40) | 0.53480 |  |
| Hypertension |  |  | 0.72540 |  |  | 0.74980 |
| No | -0.64 (-4.62, 3.34) | 0.75300 |  | -0.12 (-0.84, 0.60) | 0.74750 |  |
| Yes | -1.79 (-5.33, 1.76) | 0.32350 |  | 0.11 (-0.72, 0.93) | 0.80050 |  |
| Estradiol |  |  |  |  |  |  |
| Take drugs |  |  | 0.77450 |  |  | 0.16780 |
| No | -3.90 (-11.30, 3.51) | 0.30310 |  | -0.78 (-1.65, 0.09) | 0.07890 |  |
| Yes | -3.71 (-8.47, 1.06) | 0.12820 |  | -0.22 (-1.11, 0.68) | 0.63460 |  |
| Diabetes |  |  | 0.69080 |  |  | 0.96670 |
| No | -3.96 (-9.10, 1.18) | 0.13140 |  | -0.16(-0.73, 0.41) | 0.58060 |  |
| Yes | -5.45 (-13.63, 2.73) | 0.19220 |  | -0.23 (-1.12, 0.67) | 0.61720 |  |
| Hypertension |  |  | 0.08980 |  |  | 0.08980 |
| No | -2.24 (-6.95, 2.47) | 0.35210 |  | -0.07 (-0.90, 0.77) | 0.87430 |  |
| Yes | -5.50 (-11.55, 0.55) | 0.07510 |  | -1.53 (-2.71, -0.36) | 0.01080 |  |
| Free testosterone |  |  |  |  |  |  |
| Take drugs |  |  | 0.80460 |  |  | 0.36780 |
| No | 0.04 (0.00, 0.07) | 0.04870 |  | -0.01 (-0.02, 0.00) | 0.06320 |  |
| Yes | 0.03 (0.01, 0.05) | 0.00360 |  | -0.00 (-0.01, 0.00) | 0.11300 |  |
| Diabetes |  |  | 0.68280 |  |  | 0.35260 |
| No | 0.02 (0.00, 0.05) | 0.03180 |  | -0.00 (-0.01, 0.00) | 0.12040 |  |
| Yes | 0.04 (0.01, 0.07) | 0.01550 |  | -0.01 (-0.01, 0.00) | 0.05570 |  |
| Hypertension |  |  | 0.61700 |  |  | 0.14910 |
| No | 0.03 (0.01, 0.06) | 0.02810 |  | -0.00 (-0.00, 0.00) | 0.62910 |  |
| Yes | 0.02 (-0.00, 0.05) | 0.09540 |  | -0.01 (-0.01, -0.00) | 0.00580 |  |

β, effect size for regression; LDL-C, low density lipoprotein cholesterol; hs-CRP, high sensitive c reactive-protein; TT, total testosterone; SHBG, sex hormone binding globulin; E2, estradiol; FT, free testosterone.

**Appendix 4.1**

Interaction test

| Exposure |  | Male |  | Female |  |
| --- | --- | --- | --- | --- | --- |
| **TT** |  |  |  |  | P |
| LDL-C | Hs-CRP |  |  |  |  |
| <2.6 | <3 | Ref. |  | Ref. |  |
| >=2.6 | <3 | -1.66 (-43.54, 40.22) | 0.9381 | -1.87 (-6.77, 3.03) | 0.4536 |
| <2.6 | >=3 | 511.89 (13.37, 1010.42) | 0.0445 | -0.57 (-58.41, 57.26) | 0.9845 |
| >=2.6 | >=3 | 511.73 (15.11, 1008.36) | 0.0438 | -3.85 (-61.41, 53.71) | 0.8957 |
| P interaction |  | 0.9512 |  | 0.6548 |  |
| **SHBG** |  |  |  |  |  |
| LDL-C | Hs-CRP |  |  |  |  |
| <2.6 | <3 | Ref. |  | Ref. |  |
| >=2.6 | <3 | 1.84 (-4.22, 7.91) | 0.5510 | 3.38 (-4.41, 11.17) | 0.3954 |
| <2.6 | >=3 | 58.18 (-13.68, 130.05) | 0.1129 | -86.59 (-177.88, 4.70) | 0.0634 |
| >=2.6 | >=3 | 61.67 (-9.95, 133.30) | 0.0919 | -86.43 (-177.27, 4.41) | 0.0626 |
| P interaction |  | 0.6417 |  | 0.5176 |  |
| E2 |  |  |  |  |  |
| LDL-C | Hs-CRP |  |  |  |  |
| <2.6 | <3 | Ref. |  | Ref. |  |
| >=2.6 | <3 | 0.60 (-2.15, 3.35) | 0.6686 | 4.60 (-6.95, 16.16) | 0.4350 |
| <2.6 | >=3 | 22.00 (-10.61, 54.61) | 0.1865 | 98.94 (-37.79, 235.67) | 0.1565 |
| >=2.6 | >=3 | 22.48 (-10.02, 54.98) | 0.1756 | 106.73 (-29.38, 242.85) | 0.1247 |
| P interaction |  | 0.9409 |  | 0.6668 |  |
| **FT** |  |  |  |  |  |
| LDL-C | Hs-CRP |  |  |  |  |
| <2.6 | <3 | Ref. |  | Ref. |  |
| >=2.6 | <3 | -0.26 (-0.89, 0.36) | 0.4091 | -0.03 (-0.09, 0.02) | 0.2171 |
| <2.6 | >=3 | 4.95 (-2.50, 12.41) | 0.1931 | 0.27 (-0.37, 0.91) | 0.4154 |
| >=2.6 | >=3 | 4.62 (-2.81, 12.05) | 0.2236 | 0.24 (-0.40, 0.87) | 0.4675 |
| P interaction |  | 0.8425 |  | 0.9004 |  |

β, effect size for regression; LDL-C, low density lipoprotein cholesterol; hs-CRP, high sensitive c reactive-protein; TT, total testosterone; SHBG, sex hormone binding globulin; E2, estradiol; FT, free testosterone.

**Appendix 4.2**

Interaction of LDL-C and hs-CRP in the risk of testosterone deficiency.

| Exposure |  | Male | Female |
| --- | --- | --- | --- |
| Testosterone deficiency |  | OR (95% CI) | OR (95% CI) |
| LDL-C | Hs-CRP |  |  |
| <2.6 | <3 | Ref. | Ref. |
| >=2.6 | <3 | 0.73 (0.37, 1.42) 0.3497 | 0.84 (0.00, Inf) 1.0000 |
| <2.6 | >=3 | 0.00 (0.00, 0.10) 0.0129 | inf. (0.00, Inf) 0.9999 |
| >=2.6 | >=3 | 0.00 (0.00, 0.14) 0.0155 | inf. (0.00, Inf) 0.9999 |
| P interaction |  | 0.1372 | 0.1206 |
